# Complete genome sequence of *Pseudomonas syringae* pv. *actinidiae* strain FX219: isolated from diseased kiwifruit branches in Jiangxi, China

**DOI:** 10.1128/mra.00324-25

**Published:** 2025-07-31

**Authors:** Mingfeng Yan, Chuanxu Wan, Xiaolin Zhao, Haiyuan Li, Xingshen Li, Yang Sun, Shuijin Huang

**Affiliations:** 1 Institute of Plant Protection, Jiangxi Academy of Agricultural Sciences 205386, Nanchang, China; 2 Jiangxi Provincial Key Laboratory of Agricultural Non-point Source Pollution Control and Waste Comprehensive Utilization, Jiangxi Academy of Agricultural Sciences 205386, Nanchang, China; The University of Arizona, Tucson, Arizona, USA

**Keywords:** *Pseudomonas syringae *pv. *actinidiae*, kiwifruit, genome analysis, eastern China

## Abstract

Here, we report the complete genome sequence of *Pseudomonas syringae* pv. *actinidiae* strain FX219 isolated from diseased kiwifruit branches grown in Jiangxi province, Eastern China. The genome consists of 6,605,063 bp, with 5,989 genes and a GC content of 58.34%, providing a basis for further exploration of pathogenic factors involved in kiwifruit bacterial canker.

## ANNOUNCEMENT

The bacterial canker of kiwifruit, caused by *Pseudomonas syringae* pv. *actinidiae* (*Psa*), has become the most devastating disease in kiwifruit cultivation, threatening the industry’s development (1). The pathogen *Psa* was first reported in Shizuoka, Japan, in 1984 and has been detected in the main kiwifruit planting countries, including China, Korea, New Zealand, and European countries (2, 3). Since 2008, the disease has occurred in Jiangxi province, China. The strain described in this study was isolated from diseased kiwifruit branches grown in Fengxin County of Jiangxi province, Eastern China. Diseased stem tissues taken from the margins of the canker were sterilized with 75% ethanol, ground, and inoculated on King’s B (KB) medium agar plate and incubated at 28°C (4). Multiple single colonies were identified as *Psa* by partially amplifying and sequencing with the *psa*-specific primer pair PsaF1/PsaR2 (280 bp product) (5). These *Psa* are streaked on KB medium agar plate and incubated at 28°C for 1 day. A single colony was picked, and the bacterium was cultured overnight in KB medium broth at 28°C, 200 rpm. Glycerol stocks were prepared from this culture at −80°C. This original frozen stock was then again streaked as single colonies onto KB agar plates and picked to KB medium broth to generate a second stock culture prepared as described above. A single colony of the FX219 strain from the bacterial plate was inoculated in KB medium broth at 28°C overnight, and the bacterial suspension was centrifuged to collect bacterial cells. Here, we presented a high-quality, complete genome sequence of *Psa* strain FX219.

Genomic DNA of the FX219 strain was isolated from 28°C KB medium-grown cells using Wizard Genomic DNA Purification Kit (Promega) according to the manufacturer’s protocol without modifications and quantified by Nanodrop 2500. Then, the genomic DNA was submitted to Shanghai Majorbio Bio-Pharm Technology Co., Ltd. (Shanghai, China), for sequencing. The same whole-genome DNA extract was sequenced on both the PacBio Sequel IIe platform and Illumina Novaseq X plus platform, using SMRTbell Express Template Prep Kit 2.0 (PacBio, CA) and NEXTflex Rapid DNA-Seq (NEB, USA) for Illumina, respectively. All bioinformatics tools were used under default parameters unless specified otherwise. The Illumina short-read data in FASTQ format were processed with fastp v0.20.0 for adapter trimming and quality control (6). Nanopore long-read data were filtered using Filtlong v0.2.1, and the filtered reads were assembled by the software Canu v2.2 (7). The NCBI Prokaryotic Genome Annotation Pipeline (PGAP, v6.8) was used to annotate the genome (8). A total of 8,171,090 and 49,899 clean reads (Q20: 98.6%, Q30: 95.5%), as well as 1,233,834,590 and 418,259,095 clean bases (Q20: 98.8%, Q30: 96.0%), were obtained from Illumina and PacBio sequencing, respectively.

To confirm the taxonomic classification of the strains, average nucleotide identity (ANI) values were determined between the genomes of strains and the genomes of their respective type strains using FastANI v1.3 (9). The ANI values between strain FX219 and a New Zealand *Psa* strain ICMP18708 (GenBank: CP012179), and FX219 and a Chinese *Psa* strain Yunnan3.2 (GenBank: CP135283) were 99.94 and 99.93, respectively. Unicycler v0.4.8 was used to assemble the genome as one circular chromosome of 6,605,063 bp and no plasmids, with a sequencing depth of 63.32%–58.34% GC content. Both Illumina and PacBio reads achieved 100% genome coverage. The general genomic characteristics of FX219 are shown in Table 1. The genome of FX219 encodes four secretory systems: type I, type II, type III, and type VI, among which the type III and type VI secretion systems have been identified as essential virulence factors in the pathogenic strain of *Psa* (10). The genome sequence provided here will help further provide a theoretical basis for the potential pathogenic factors responsible for kiwifruit bacterial canker.

**TABLE 1:** Genome features of *Pseudomonas syringae* pv. *actinidiae* strain FX219

Sample name	FX219
Genome size (bp)	6,605,063
Chromosome number	1
GC content (%)	58.34
Genes	5,989
N_50_	8,398
tRNAs	65
sRNA	196
ncRNA	4
(5s,16s,23s) rRNAs	(6, 5 ,5)
Number of prophage	322
Type I secretion system genes	3
Type II secretion system genes	13
Type III secretion system genes	22
Type VI secretion system genes	30

## Data Availability

The FX219 genome sequence was deposited in the GenBank database under accession number CP186534, BioSample number SAMN47739214, BioProject number PRJNA1245049, and Sequence Read Archive (SRA) accession number SRR32962833 and SRR34348544, respectively.

## References

[1] Vanneste JL. 2017. The scientific, economic, and social impacts of the New Zealand outbreak of bacterial canker of kiwifruit (Pseudomonas syringae pv. actinidiae). Annu Rev Phytopathol 55:377–399. doi:10.1146/annurev-phyto-080516-035530 28613977

[2] Serizawa S, Ichikawa T, Takikawa Y, Tsuyumu S, Goto M. 1989. Occurrence of bacterial canker of kiwifruit in Japan: description of symptoms, isolation of the pathogen and screening of bactericides. Jpn J Phytopathol 55:427–436. doi:10.3186/jjphytopath.55.427

[3] Chapman JR, Taylor RK, Weir BS, Romberg MK, Vanneste JL, Luck J, Alexander BJR. 2012. Phylogenetic relationships among global populations of Pseudomonas syringae pv. actinidiae. Phytopathology 102:1034–1044. doi:10.1094/PHYTO-03-12-0064-R 22877312

[4] Vanneste JL, Cornish DA, Yu J, Stokes CA. 2014. First report of Pseudomonas syringae pv. actinidiae the causal agent of bacterial canker of kiwifruit on Actinidia arguta Vines in New Zealand. Plant Dis 98:418. doi:10.1094/PDIS-06-13-0667-PDN 30708440

[5] Li L, Pan H, Deng L, Feng D, Zhong C. 2021 First report of bacterial leaf spot disease of Broussonetia papyrifera caused by Pseudomonas syringae pv. actinidiae in China. Plant Dis 105:696. doi:10.1094/PDIS-07-20-1527-PDN

[6] Chen S, Zhou Y, Chen Y, Gu J. 2018. Fastp: an ultra-fast all-in-one FASTQ preprocessor. Bioinformatics 34:i884–i890. doi:10.1093/bioinformatics/bty560 30423086 PMC6129281

[7] Koren S, Walenz BP, Berlin K, Miller JR, Bergman NH, Phillippy AM. 2017. Canu: scalable and accurate long-read assembly via adaptive k-mer weighting and repeat separation. Genome Res 27:722–736. doi:10.1101/gr.215087.116 28298431 PMC5411767

[8] Tatusova T, DiCuccio M, Badretdin A, Chetvernin V, Nawrocki EP, Zaslavsky L, Lomsadze A, Pruitt KD, Borodovsky M, Ostell J. 2016. NCBI prokaryotic genome annotation pipeline. Nucleic Acids Res 44:6614–6624. doi:10.1093/nar/gkw569 27342282 PMC5001611

[9] Jain C, Rodriguez-R LM, Phillippy AM, Konstantinidis KT, Aluru S. 2018. High throughput ANI analysis of 90K prokaryotic genomes reveals clear species boundaries. Nat Commun 9:5114. doi:10.1038/s41467-018-07641-9 30504855 PMC6269478

[10] Wang Q, Zhang Y, Chen R, Zhang L, Fu M, Zhang L. 2024. Comparative genomic analyses provide insight into the pathogenicity of three Pseudomonas syringae pv. actinidiae strains from Anhui Province, China. BMC Genomics 25:461. doi:10.1186/s12864-024-10384-1 38734623 PMC11088785

